# Similar 5F-APINACA Metabolism between CD-1 Mouse and Human Liver Microsomes Involves Different P450 Cytochromes

**DOI:** 10.3390/metabo12080773

**Published:** 2022-08-22

**Authors:** Samantha V. Crosby, Izzeldin Y. Ahmed, Laura R. Osborn, Zeyuan Wang, Mary A. Schleiff, William E. Fantegrossi, Swati Nagar, Paul L. Prather, Gunnar Boysen, Grover P. Miller

**Affiliations:** 1 Department of Biochemistry and Molecular Biology, University of Arkansas for Medical Sciences, Little Rock, AR 72205, USA; 2 Department of Chemistry and Physics, Department of Biological Sciences, Arkansas State University, Jonesboro, AR 72401, USA; 3 Department of Pharmaceutical Sciences, Temple University, Philadelphia, PA 19122, USA; 4 Department of Pharmacology and Toxicology, University of Arkansas for Medical Sciences, Little Rock, AR 72205, USA; 5 Department of Environmental and Occupational Health, University of Arkansas for Medical Sciences, Little Rock, AR 72205, USA

**Keywords:** synthetic cannabinoid, 5F-APINACA, 5F-AKB48, P450, metabolism, enzyme kinetics, drug abuse, CB1 receptor, human, mouse

## Abstract

In 2019, synthetic cannabinoids accounted for more than one-third of new drugs of abuse worldwide; however, assessment of associated health risks is not ethical for controlled and often illegal substances, making CD-1 mouse exposure studies the gold standard. Interpretation of those findings then depends on the similarity of mouse and human metabolic pathways. Herein, we report the first comparative analysis of steady-state metabolism of *N*-(1-adamantyl)-1-(5-pentyl)-1*H*-indazole-3-carboxamide (5F-APINACA/5F-AKB48) in CD-1 mice and humans using hepatic microsomes. Regardless of species, 5F-APINACA metabolism involved highly efficient sequential adamantyl hydroxylation and oxidative defluorination pathways that competed equally. Secondary adamantyl hydroxylation was less efficient for mice. At low 5F-APINACA concentrations, initial rates were comparable between pathways, but at higher concentrations, adamantyl hydroxylations became less significant due to substrate inhibition likely involving an effector site. For humans, CYP3A4 dominated both metabolic pathways with minor contributions from CYP2C8, 2C19, and 2D6. For CD-1 mice, Cyp3a11 and Cyp2c37, Cyp2c50, and Cyp2c54 contributed equally to adamantyl hydroxylation, but Cyp3a11 was more efficient at oxidative defluorination than Cyp2c members. Taken together, the results of our in vitro steady-state study indicate a high conservation of 5F-APINACA metabolism between CD-1 mice and humans, but deviations can occur due to differences in P450s responsible for the associated reactions.

## 1. Introduction

In 2019, synthetic cannabinoid (SCB) receptor agonists accounted for more than one-third of new drugs of abuse worldwide [1]. SCBs interact with cannabinoid type 1 receptor (CB1R) to induce a “high” similar to cannabis, yet, unlike cannabis, chronic or acute SCB use may cause numerous and severe adverse drug reactions, including persistent psychosis, tachycardia, cardiac arrhythmia and myocardial infarction, seizures and convulsions, and even death [2]. SCB users are 30-fold more likely to require emergency medical treatment than users of plant cannabis [3]. The assessment of these health risks for SCBs is critical for scaling health risks and establishing regulatory guidelines. Nevertheless, controlled clinical studies are not feasible for SCBs due to unacceptable health risks and ethical concerns. Moreover, under-reporting and incomplete reporting of adverse outcomes associated with SCBs are common due to legal liabilities and social stigma for users. These practical issues confound the ability to accurately assess the impact of SCBs on human health; thus, exposure studies with CD-1 mice have become the gold standard in behavioral and toxicological fields to extrapolate effects to humans. 

Since the early 1980s, CD-1 mice have been among the most widely used outbred strains in toxicology research [4], and continue to provide validated models of tolerance to the effects of numerous drugs of abuse, including opioids [5], ethanol [6], benzodiazepines [7], inhalants [8], and caffeine [9]. In addition, CD-1 mice are also widely used in the study of drug dependence and withdrawal [10,11,12], rewarding effects [13,14,15], and drug-elicited toxicities [16]. Recently, CD-1 mice have been widely used to examine the behavioral and toxicological effects of abused synthetic cannabinoids, including JWH-018 [17,18], 5F-ADBINACA, AB-FUBINACA, and STS-135 [19], AKB48 and 5F-AKB48 [20], and JWH-250 and JWH-073 [21]. Evaluation of cannabimimetic effects in rodents typically involves a “tetrad” assay with measures of hypothermia, antinociception, catalepsy, and suppression of locomotor activity [22]. Each endpoint is dose-dependently elicited by CB1R agonists and attenuated by CB1R antagonists using hypothermia as the hallmark of CB1R activation [23,24]. Previously, we reported tolerance development to tetrad effects for several SCBs, and discovered initial evidence that hypothermia tolerance was mediated by dramatic downregulation and desensitization of CB1Rs in mice [25]. The translatability of these findings to humans depends on the conservation of SCB metabolic pathways because these processes alter drug structure, and hence the potency and overall clearance of the drugs, impacting the strength and longevity of the SCB response. Accordingly, suppression of SCB metabolism by the pan-cytochrome P450 (CYP) inhibitor 1-aminobenzotriazole (ABT) increased hypothermia in mice caused by numerous SCBs, including 5F-APINACA (5F-AKB48) [26,27]. These studies implicated a common dominance of CYPs in metabolism between mice and humans; however, there are no current studies that determine which metabolic pathways exist in mice and how important they are in the metabolic clearance of SCBs relative to humans. This lack of knowledge raises questions as to the suitability of CD-1 mice as appropriate surrogates for understanding the contributions of SCB metabolism to responses and the factors influencing these responses with respect to humans. 

**Figure 1:**
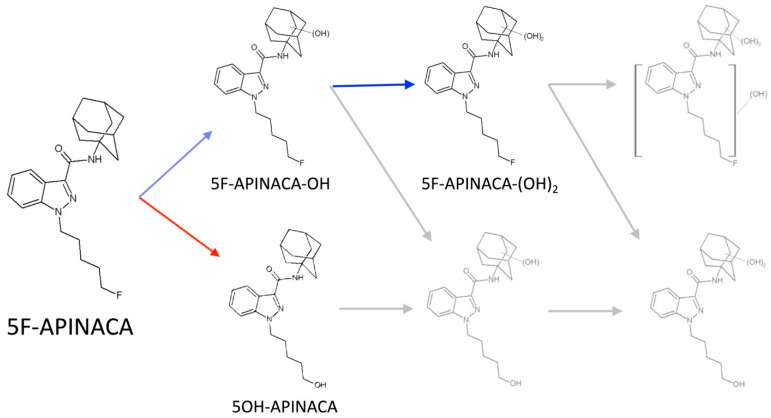
Reported competing and intersecting metabolic pathways of 5F-APINACA. Current experimental design limited metabolism to sequential oxidation of the adamantyl group (blue arrows) and the initial oxidative defluorination (red arrows), avoiding previously reported steps and higher order metabolites, shown in gray [27,28,29,30].

Herein, we describe the first assessment and comparison of the metabolism of SCB, 5F-APINACA, by human and CD-1 mouse liver microsomes for in vitro steady-state analyses. As a first step, we further optimized our previously reported conditions [28] to minimize the turnover of highly efficient reactions for a more accurate, direct assessment of the initial committed steps for 5F-APINACA oxidations. Unlike previous reports [27,28,29,30], 5F-APINACA was limited to the sequential oxidation of the adamantyl group and initial oxidative defluorination, avoiding reaction steps and higher order metabolites (Figure 1). These initial reaction studies were coupled with liquid chromatography–mass spectrometry (LC–MS) analyses to characterize metabolite structures and infer their identities. Under steady-state conditions, we determined kinetic profiles for human and CD-1 microsomal metabolism of 5F-APINACA. As a first approach, traditional kinetic models were fit to the data for the comparison of apparent mechanisms and kinetic parameters to those reported in the literature. Follow up analyses involved more in-depth kinetic assessments of individual reaction steps and the global fitting of differential rate equations to data for all metabolites, simultaneously [31,32]. These efforts identified which enzyme–substrate interactions played critical roles in determining the overall relationship between substrate concentration and rates of reactions. Lastly, we assessed the conservation of CYP isozymes responsible for metabolism between species using chemical inhibitor phenotyping.

## 2. Materials and Methods

### 2.1. Materials

All chemical solvents, salts, and buffers were purchased from Thermo Scientific (Waltham, MA, USA). CYP isozyme inhibitors α-naphthoflavone, (+)-*N*-3-benzylnirvanol, ketoconazole, montelukast sodium, quinidine, sulfaphenazole, ticlopidine hydrochloride, and tranylcypromine sulfate were purchased from Millipore-Sigma. The substrate (5OH-APINACA) and CYP isozyme inhibitors (1-aminobenzotriazole and 4-methylpyrazole hydrochloride) were purchased from Cayman Chemical (Ann Arbor, MI, USA). NADPH-regenerating system components NADP disodium salt, glucose-6-phosphate dehydrogenase, and glucose-6-phosphate were purchased from Millipore-Sigma (St. Louis, MO, USA), while magnesium chloride salt was purchased from Thermo Fisher Scientific. The substrate 5F-APINACA was provided by Dr. William Fantegrossi. Human liver microsomes pooled from 150 individuals, lot#38296, and P450 contribution of 0.380 nmol/mg, were purchased from Corning (Corning, NY, USA). CD-1 male mouse liver microsomes pooled from 6424 mice, lot#2010017, and P450 contribution of 0.671 nmol/mg, were purchased from Sekisui Xenotech (Kansas City, KS, USA). Internal standard acenocoumarol was purchased from Toronto Research Chemicals (Toronto, ON, Canada). ACD/ChemSketch 2017.2.1 software (Toronto, ON, Canada) was used for rendering structures of molecules. 

### 2.2. Steady-State Kinetics for 5F-APINACA Metabolism by Human and Mouse Liver Microsomes

The in vitro 5F-APINACA analyses relied on reactions with human liver microsomes 150 (HLM150) as a model for the average adult human liver and CD-1 mouse liver microsomes as the standard for SCB studies. In a 96-half-well microplate, reaction conditions included human or mouse liver microsomes in 100 mM potassium phosphate buffer pH 7.4, 0.1% methanol (co-solvent), and 5F-APINACA concentrations ranging from 0 to 250 µM. The microplates were preincubated for 15 min at 37 °C with shaking at 350 rpm using a BMG Labtech THERMOstar incubator (Ortenberg, Germany). The addition of an NADPH-regenerating system (4 U/mL glucose-6-phosphate dehydrogenase, 12 mM glucose 6-phosphate, 12 mM MgCl_2_, 4 mM NADP^+^) initiated the reaction. Reactions were quenched by adding an equal volume of ice-cold acetonitrile containing an internal standard (2 μM acenocoumarol final). Samples were chilled on ice for 10 min to optimize precipitation of proteins in phosphate buffer [33]. After 3500 rpm centrifugation at 4 °C for 15 min using a Sorvall ST 16R centrifuge (Thermo Scientific; Waltham, MA, USA), the supernatant was transferred to HPLC vials for HPLC analyses. Standards for metabolite quantifications were prepared with human or mouse liver microsomes in 100 mM potassium phosphate buffer pH 7.4, 0.1% methanol (co-solvent), and 5OH-APINACA concentrations ranging from 0 to 15 µM. The microplates were preincubated for 15 min at 37 °C with shaking at 350 rpm. Reactions were initiated upon addition of the NADPH-regenerating system or 100 mM potassium phosphate as a negative control. For kinetic studies, initial control experiments were carried out to determine the lowest protein concentration and optimal reaction time within the linear response range for all observed metabolites reflecting steady-state conditions. Each set of steady-state reactions were performed at least in triplicate and replicated two times at a minimum. The composition of reactions was determined by liquid chromatographic analysis involving detection of analytes via UV/visible absorbance and mass spectroscopy (Section 2.5).

### 2.3. Kinetic Analyses with Explicit Equations and Numerical Methods

We employed explicit equations and numerical methods to model kinetic profiles. In the first case, metabolite levels were used to calculate initial reaction rates for plotting against substrate concentration with GraphPad Prism 9.2 from GraphPad Software, Inc. (San Diego, CA, USA). Multiple traditional kinetic models (Michaelis–Menten, substrate inhibition, and Hill cooperativity) were fit to the kinetic data. For the numerical methods, ordinary differential equations (ODEs) describing single-substrate (ES) and multi-substrate (ESS) binding kinetics [32] were initially used to fit the models to the primary metabolite formation datasets. The AICc [34] was used to determine the most appropriate model for the same dataset. Predicted parameter estimates from the most appropriate model were then used as initial parameter estimates for a variety of enzyme–substrate–sequential metabolism (ESP1P2P3) models. Schemes included fast- and slow-release rates of metabolite P2 from the enzyme site, either after the first binding event, the second binding event, or both events. A final best-fit model was selected based on AICc values, weighted residuals, and parameter estimate errors. For comparison of all multi-substrate-sequential metabolism models, including the final integrated model, all metabolite data were used simultaneously for model fitting. All association rate constants were fixed at 270 μM^−1^ min^−1^. When any rate constant within the model had to be held constant, a sensitivity analysis was performed over a range of values to ensure the validity of the results. Based on preliminary model fitting attempts, consistent enzyme kinetic schemes were developed for fitting to both human and CD-1 mouse liver microsomal reaction datasets. Mathematica version 12.3 (Wolfram Research, Champaign, IL, USA) was used for model fitting. The ODEs were numerically solved with NDSolve to provide interpolated functions of each enzyme species. The NonlinearModelFit function was used to parameterize the rate constants with 1/Y weighting. Specific properties were assigned, including MaxSteps→100,000 and PrecisionGoal→infinity(∞) for NDSolve.

### 2.4. Chemical Inhibitor Phenotyping of Reactions for CYP Contributions

Inhibitor phenotyping experiments [35] were carried out to qualitatively identify probable P450 isozymes catalyzing 5F-APINACA metabolic pathways present in our mouse and human liver microsome studies. Reactions contained 0.1 mg/mL protein (MLM or HLM), 25 µM 5F-APINACA, specific CYP inhibitors, 0.25% methanol, 1% acetonitrile, 1.0 mM NADPH-regenerating system, and 100 mM phosphate buffer, pH 7.4. The concentrations and choice of the inhibitors were based on existing literature demonstrating selective bias in inhibiting specific P450 isozymes. For each experiment, the following were included as final concentrations: 10 µM furafylline for CYP1A2, 2 µM tranylcypromine (TCP) for CYP2A6, 3 µM ticlopidine (TIC) for CYP2B6, 16 µM montelukast (MTK) for CYP2C8, 10 µM sulfaphenazole (SPA) for CYP2C9, 16 µM (+)-*N*-3-benzylnirvanol (NBZ) for CYP2C19, 2 µM quinidine (QND) for CYP2D6, 30 µM 4-methylpyrazole (4MP) for CYP2E1, 1 µM ketoconazole (KCZ) for CYP3A4, and 1000 µM ABT for all CYPs. Each inhibitor stock solution (except FUR) was prepared in potassium phosphate buffer pH 7.4 with 1% acetonitrile (final) as a co-solvent. After a preincubation period of 15 min with shaking, the reactions were initiated with the addition of a NADPH-regenerating system and shaken. For FUR, the inhibitor was prepared in methanol and a microsomal reaction was carried out at 10 µM for 10 min prior to addition of 5F-APINACA. After 30 min, all reactions were quenched, processed, and analyzed by HPLC as previously described. The resultant values were normalized to reaction rates observed in absence of inhibitors to yield a percent inhibition value.

### 2.5. HPLC Resolution and Analysis of 5F-APINACA Analytes from Reactions

Sample reactions were analyzed to quantitate metabolites by absorbance. Analytes were separated on a Waters XBridge BEH C18 3.5 μM column (4.6 mm × 100 mm) using an Agilent 1100 series HPLC instrument equipped with a VWD detector (280 nm). The mobile phase consisted of solvent A (10% solvent B, 90% water) and solvent B (0.1% formic acid/acetonitrile). A gradient method started at 80% solvent A for the first minute, then decreased to 70% for the next 6 min. The method held 70% A for 3 min before decreasing to 15% A over 11 min, then returning to 80% over 3 min and holding for the final 2 min. The flow rate was 1.2 mL/min for a total run time of 25 min. Parent drug and metabolite responses were normalized to the internal standard acenocoumarol and quantitated using a standard curve generated with 5OH-APINACA. The absorbance response corresponds to the indazole ring, which remained unmodified in these reactions [28], so that the relative absorbance response for all analytes were approximately the same, making inference for quantitation purposes possible.

### 2.6. Statistical Analyses

Various statistical approaches were employed among the experimental studies to identify the most likely models for explaining the data or establish the significance of the findings. For the explicit kinetic models, the best-fit kinetic model and corresponding constants were determined using the extra sum-of-squares F test. In addition, we excluded statistically preferred mechanisms when best-fit values possessed open confidence intervals. For the numerical models, the AICc [34] was used to determine the most appropriate model for the same dataset. Lastly, statistical analyses for the phenotyping studies were performed by comparing reactions containing chemical inhibitors to negative controls only containing co-solvent using the student *t*-test analysis.

## 3. Results

### 3.1. Conditions Were Optimized to Improve Accuracy of Kinetic Measures for Microsomal 5F-APINACA Metabolism

The contributions of multiple metabolic steps and intersecting pathways confounds the analysis of individual metabolic reactions between the species. Consequently, we confirmed the linearity of rates as a function of protein concentration and time to ensure steady-state conditions (data not shown), and then selected a protein concentration with minimal, but measurable, 5F-APINACA turnover. These experiments replicated metabolite profiles based on chromatographic separation and MS characterization using parent *m*/*z* and mass transitions, as reported previously by ourselves [27,28] and others [29,30]. At 0.1 mg/mL protein for human and mouse reactions, there were five metabolites detectable by highly sensitive LC–MS methods, but only three were quantifiable by liquid chromatographic methods coupled with absorbance detection (LC–UV/Vis). The latter analyses showed evidence for 5F-APINACA oxidative defluorination to yield 5OH-APINACA, and oxidation of the adamantyl group to subsequently yield mono- and dihydroxylated metabolites, i.e., 5F-APINACA-OH and 5F-APINACA-(OH)_2_, respectively. The remaining secondary and tertiary metabolites were undetectable by absorbance, and thus, were produced at rates less than 10 pmol/min/mg protein, making their contributions insignificant in steady-state reactions.

### 3.2. Human 5F-APINACA Metabolism Favored Two Competing Pathways Similarly

**Figure 2:**
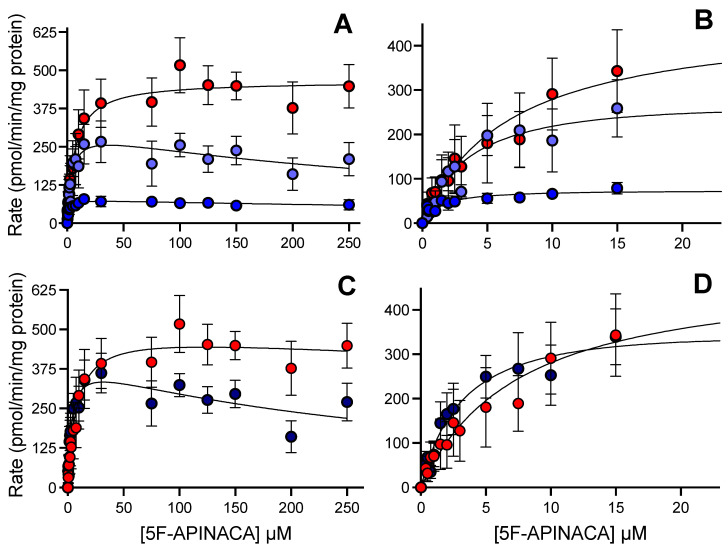
Steady-state metabolism 5F-APINACA by human liver microsomes fit to traditional kinetic models. (**A**) Kinetic plot for primary metabolites, i.e., 5OH-APINACA from oxidative defluorination of the pentyl group (red), and 5F-APINACA-OH from monohydroxylation of the adamantyl group (light blue), as well as the secondary metabolite, 5F-APINACA-(OH)_2_ from another hydroxylation of the adamantyl group (blue). (**B**) Same data and model fits from (**A**) highlighting kinetics at lower 5F-APINACA concentrations. (**C**) Kinetic data from common adamantyl hydroxylation pathway were combined and replotted (dark blue) for comparison to 5OH-APINACA kinetics. (**D**) Same data and model fits from (**C**) highlighting kinetics at lower 5F-APINACA concentrations. Steady-state reaction conditions and data analyses were carried out as described in the Materials and Methods. Each data point is an average of 12 replicates, and the displayed curve reflects the best-fit model for the data. The corresponding mechanisms and constants are reported in Table 1.

The 5F-APINACA metabolism of pooled human liver microsomes led to two pathways with distinct steady-state kinetic mechanisms (Table 1). The two successive hydroxylations of the adamantyl group led to substrate inhibition kinetic profiles with very low K_m_s, followed by very high K_i_s (Figure 2A). We then combined the observed rates for these coupled reactions and assessed the overall kinetics for adamantyl group hydroxylation (Figure 2C). By contrast, the formation kinetics of 5OH-APINACA fit best to a Michaelis–Menten mechanism (Figure 2A) with the highest observed V_max_ and K_m_ values, respectively. When compared, the specificity for oxidation of the adamantyl group expressed by V_max_/K_m_ or k_cat_/K_m_ [36] exceeded that for oxidative defluorination. Nevertheless, substrate inhibition of the combined hydroxylations of the adamantyl group resulted in comparable initial rates of turnover between the competing pathways at low substrate concentrations (<15 µM), and the dominance of the pathway for 5OH-APINACA at high concentrations (>50 µM) (Figure 2B,D).

### 3.3. Human 5F-APINACA Microsomal Metabolism Impacted by Substrate Effector Site

**Figure 3:**
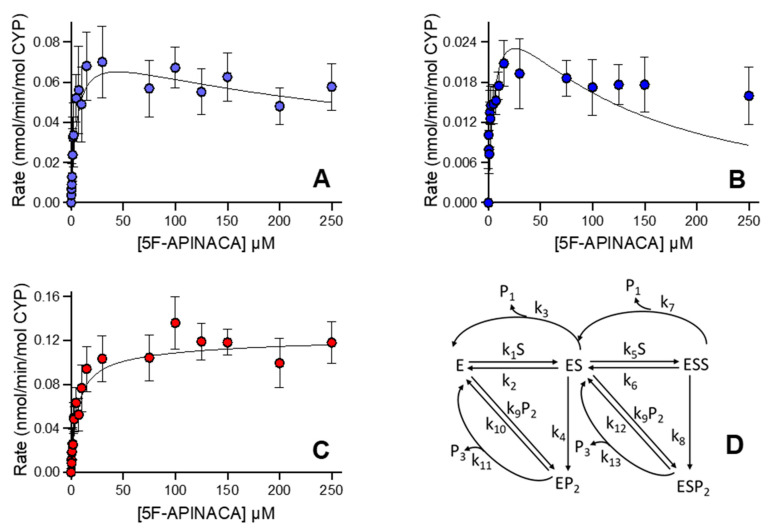
Steady-state metabolism 5F-APINACA by human liver microsomes fit to differential rate equations. Kinetic plots for 5F-APINACA-OH (**A**), 5F-APINACA-(OH)_2_ (**B**), and 5OH-APINACA (**C**) from Figure 2 were refit globally to differential equations for individual rate constants for the mechanism shown in (**D**) and described in the Materials and Methods. In the kinetic scheme, P1 denotes 5OH-APINACA, P2 denotes 5F-APINACA-OH, and P3 denotes 5F-APINACA-(OH)_2_. Each data point is an average of 12 replicates, and corresponding constants are reported in Table 2.

We reanalyzed the kinetic profiles for human microsomal reactions to better understand how individual reaction steps determined the relationship between observed initial rates and 5F-APINACA concentration using differential equations and global treatment of the data. Rather than total protein concentration, initial rates were normalized to total CYP concentration (0.38 μM) present in this batch of human liver microsomes. Kinetic schemes were developed based on the metabolic pathways shown in Figure 1. The kinetic schemes assumed that only one CYP enzyme was responsible for substrate metabolism to develop respective differential rate equations. When fitting single-substrate (ES) models and multi-substrate (ESS) models to primary metabolite formation data, multi-substrate models showed a better fit with lower Akaike Information Criterion (AICc) values than ES models (data not shown). The formation of 5OH-APINACA (P1) exhibited multi-substrate kinetics with the assumption of a fast release of the product from the enzyme active site. This base model was expanded to include the formation of 5F-APINACA-OH (P2) and its sequential conversion to 5F-APINACA-(OH)_2_ (P3), resulting in several multi-substrate sequential metabolism models, including various schemes for fast vs. slow metabolite release of 5F-APINACA-OH (P2). The best model was selected based on objective functions as described in the Materials and Methods. Slow metabolite release models for P2, assuming the formation of the EP2 and ESP2 complex (Figure 3D), resulted in a lower AICc value when compared to other schemes for slow release (e.g., no EP2 formation, AICc = −513). The final selected model (Figure 3D) includes two branched pathways from EP2 and ESP2, respectively. This schema necessitated parameter values for k_11_ and k_13_ to be fixed to 40 min^−1^. The corresponding K_m_, V_max_, and V_max_/K_m_ were calculated from parameter estimates of micro-rate constants (Table 2). Sensitivity analysis was performed to test the stability of the model. The fixed k_11_ and k_13_ values were varied within the range of 1 to 60 min^−1^, and similar (k_10_ + k_11_)/k_9_ and (k_12_ + k_13_)/k_9_ values were obtained. For initial estimates of all other model parameters, either increasing or decreasing the initial estimate twofold resulted in similar results.

### 3.4. CD-1 Mouse 5F-APINACA Microsomal Metabolism Favored the Same Two Competing Pathways

**Figure 4:**
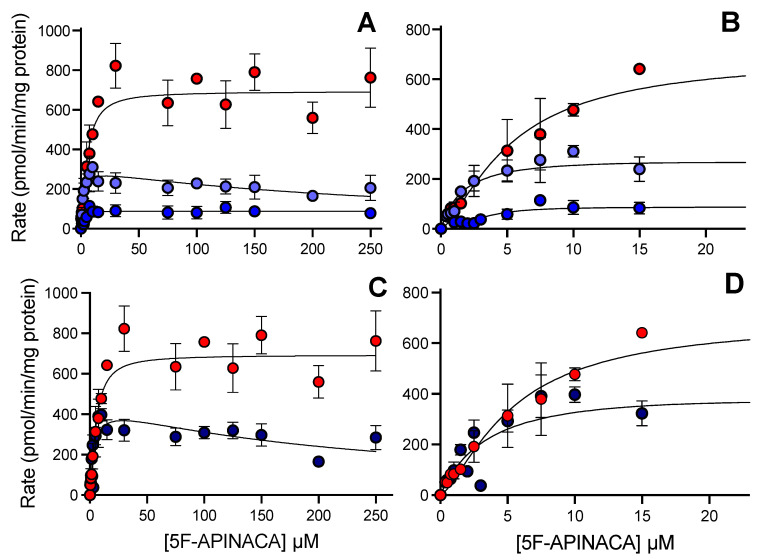
Steady-state metabolism 5F-APINACA by CD-1 mouse liver microsomes fit to traditional kinetic models. (**A**) Kinetic plot for primary metabolites, i.e., 5OH-APINACA from oxidative defluorination of the pentyl group (red), and 5F-APINACA-OH from monohydroxylation of the adamantyl group (light blue), as well as the secondary metabolite, 5F-APINACA-(OH)_2_ from another hydroxylation of the adamantyl group (blue). (**B**) Same data and model fits from (**A**) highlighting kinetics at lower 5F-APINACA concentrations. (**C**) Kinetic data from common adamantyl hydroxylation pathway were combined and replotted (dark blue) for comparison with 5OH-APINACA kinetics. (**D**) Same data and model fits from (**C**) highlighting kinetics at lower 5F-APINACA concentrations. Steady-state reaction conditions and data analyses were carried out as described in the Materials and Methods. Each data point is an average of 12 replicates, and the displayed curve reflects the best-fit model for the data. The corresponding mechanism and constants are reported in Table 2.

As observed for humans, 5F-APINACA metabolism by CD-1 mouse liver microsomes led to two pathways with distinct steady-state kinetic mechanisms (Table 3). The initial hydroxylation of the adamantyl group led to substrate inhibition, while the second hydroxylation favored a highly positive cooperativity (Hill) mechanism (Figure 4A), suggesting a less-coupled process than observed with human liver microsomes. Both mouse reactions still involved high-affinity substrate interactions and rates of turnover. The combination of the rates for the overall pathway kinetics led to a preference for the substrate inhibition mechanism (Figure 4C). The oxidative defluorination reaction showed weakly positive cooperative kinetics (Figure 4A), and the highest kinetic values. Despite differences among individual reaction constants, the corresponding specificities were similar for the two metabolic pathways. Relatively even competition between them is evident from the highlighted rates at low (<15 μM), pharmacologically relevant 5F-APINACA levels, presented in Figure 4D.

### 3.5. CD-1 Mouse 5F-APINACA Microsomal Metabolism Impacted Similarly by Substrate Effector Site

**Figure 5:**
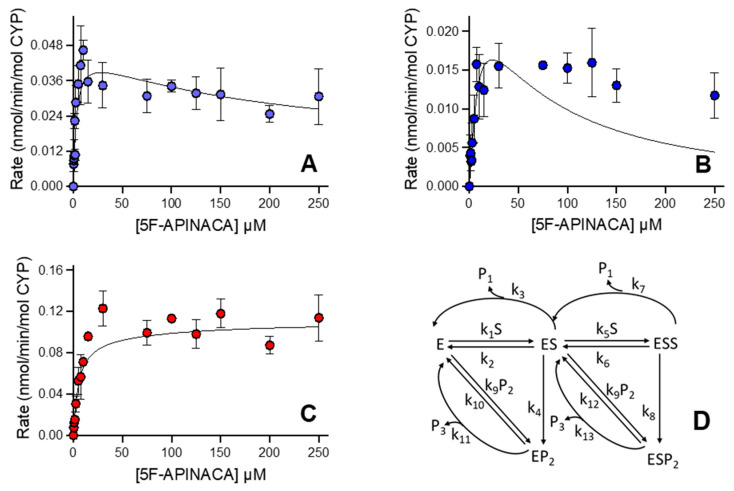
Steady-state metabolism of 5F-APINACA by CD-1 mouse liver microsomes fit to differential rate equations. Kinetic plots for 5F-APINACA-OH (**A**), and 5F-APINACA-(OH)_2_ (**B**), and 5OH-APINACA (**C**) from Figure 4 were refit globally to differential equations for individual rate constants for the mechanism shown in (**D**) and described in Materials and Methods. Each data point is an average of 12 replicates, and corresponding constants reported in Table 4.

The follow up kinetic analyses with differential equations mirrored the same two substrate-binding mechanisms ascribed to human microsomal metabolism of 5F-APINACA. As a first step, initial rates were normalized to total CYP concentration (0.67 μM) present in this batch of CD-1 mouse liver microsomes. The substrate binding characteristics of the mouse vs. human CYP isoform were assumed to be similar [37]. As conducted in the human models, several schemes were compared, and the best model was selected for the mouse liver microsomal data based on objective functions, as described in the Materials and Methods. The model (Figure 5D) including EP2 and ESP2 complexes was selected as the best one for the MLM dataset. The k_11_ and k_13_ values were fixed at 5 min^−1^, and the corresponding K_m_, V_max_, and V_max_/K_m_ values calculated from estimated micro-rate constants are listed in Table 4. Sensitivity analysis was performed to test the stability of the model. Similar (k_10_ + k_11_)/k_9_ and (k_12_ + k_13_)/k_9_ values were obtained when varying the k_11_ and k_13_ value from 2.5 to 5 min^−1^. For initial estimates of all other model parameters, either increasing or decreasing the initial estimates twofold resulted to similar estimates.

### 3.6. CYP Selectivity and Specificity toward 5F-APINACA Differ between Humans and CD-1 Mice

**Figure 6:**
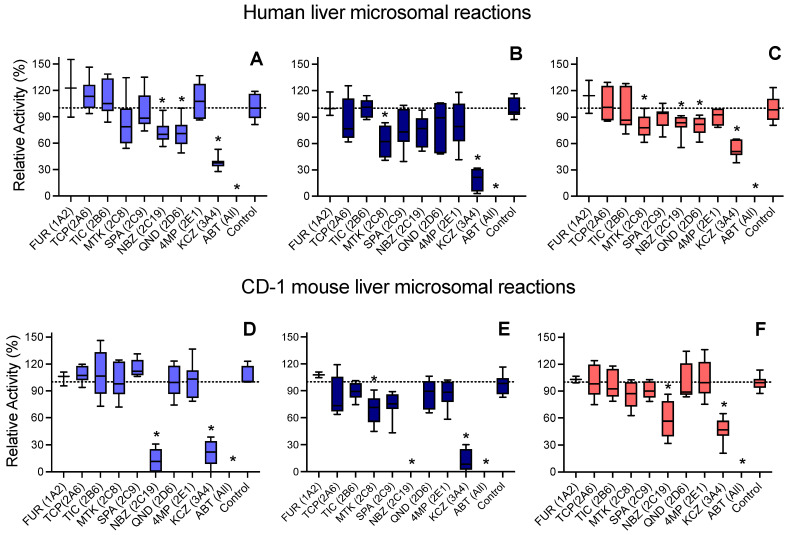
Phenotypic chemical inhibitors used to implicate individual CYPs carrying out observed 5F-APINACA reactions. Human liver microsomal reactions at 25 µM 5F-APINACA were blocked from generating 5F-APINACA-OH (**A**), 5F-APINACA-(OH)_2_ (**B**), and 5OH-APINACA (**C**) using inhibitors specific for human CYPs, indicated in parentheses. CD-1 mouse liver microsomal reactions at 25 µM 5F-APINACA were blocked from generating 5F-APINACA-OH (**D**), 5F-APINACA-(OH)_2_ (**E**), and 5OH-APINACA (**F**) using inhibitors specific for human CYPs, indicated in parentheses. Rates were normalized to those of the inhibited controls and displayed as an average of 6 replicates. Significance indicated by *, for which *p* < 0.05, as described in the Materials and Methods.

As a complement to microsomal reactions, we carried out chemical inhibitor phenotyping studies to determine which CYPs were responsible for undergoing reactions with human and CD-1 mouse liver microsomes. Introduction of the pan-CYP inhibitor ABT to human microsomal reactions completely blocked the formation of all metabolites, demonstrating the sole contribution of this enzyme class to the observed oxidations (Figure 6A–C). For both pathways, CYP3A4 was the dominant isozyme based on the inhibition of these pathways using the CYP3A4 inhibitor ketoconazole, while other inhibitors indicated minor contributions by CYP2C8, 2C19, and 2D6 to metabolism. Studies carried out with CD-1 microsomes recapitulated the sole role of CYPs in 5F-APINACA metabolism (Figure 6D–F). Moreover, Cyp3a11, the mouse ortholog of CYP3A4 [37], was the dominant isozyme carrying out metabolic reactions. Interestingly, the inhibitor for CYP2C19 also implicated Cyp2c37, Cyp2c50, and Cyp2c54 [37] as other dominant contributors to adamantyl hydroxylation, but minor contributors to oxidative defluorination.

## 4. Discussion

### 4.1. Experimental Design Impacts Metabolism Observations and Conclusions

Our goal was to experimentally assess the capacity of CD-1 mouse microsomes to replicate human reactions toward 5F-APINACA. In so doing, our findings highlight two important experimental design qualities requiring optimization for conducting more accurate and interpretable assessments. First, protein concentration in human microsomes played a role in determining the relative importance of competing metabolic pathways. The current reactions at 0.1 mg/mL protein recapitulated the contribution of two metabolic pathways for 5F-APINACA, as reported previously for reactions at 0.25 [28] and 1.0 [27] mg/mL protein. Nevertheless, there was a key difference. At higher protein concentrations, the specificity for oxidative defluorination was far greater than that for adamantyl hydroxylation, yet these values were comparable at 0.1 mg/mL protein in this study. This difference likely reflects the subsequent consumption of adamantyl-hydroxylated metabolites based on higher order metabolites observed at higher protein concentrations. Under these conditions, the apparent kinetics of adamantyl hydroxylation would then be less reliable than the kinetics we currently report at 0.1 mg/mL protein and use for the comparison of kinetics between species. Second, the traditional normalization of microsomal reactions to protein concentration can mask actual species differences, wherein the respective CYP content does not necessarily correlate with protein concentration. In our case, the observed twofold higher rates of mouse metabolism of 5F-APINACA over those exhibited by the human liver microsomes were not due to more efficient metabolism. Rather, this finding presumably reflects an approximate twofold higher CYP content in the mouse microsomal preparation (0.67 μM) than for the human microsomal preparation (0.38 μM). Importantly, this difference in content is not uniform; a cursory analysis of the CYP-to-protein content among different batches of human and mouse microsomes showed a more than twofold variability. In following studies, the choice of microsomes could determine whether species differences are observable or not when normalizing kinetics to total protein rather than CYP content, where CYP is the main determinant of metabolism. While these issues impacted the current study, they would apply to any analysis of highly specific metabolic processes involving competing and sequential reactions, as is commonly observed for SCBs and other drugs.

### 4.2. Human and CD-1 Mice Share Common Competing Metabolic Pathways for 5F-APINACA

The comparison of 5F-APINACA metabolism by human and CD-1 mouse liver microsomes showed conservation of competing metabolic pathways for the two species. In fact, adamantyl hydroxylation and oxidative defluorination were equally competitive in metabolism. This competition may be relevant to both pharmacological activity of the drug and overall drug clearance. Oxidative defluorination yields 5OH-APINACA, which undergoes subsequent oxidation to an aldehyde and then an acid [27,28,29,30]. The carboxylic acid metabolite is much less active toward CB1R than the parent drug, and more susceptible to elimination as a glucuronide [27,29], resulting in detoxification. Despite similar competing metabolic pathways, there were subtle differences in the reaction kinetics between humans and mice (Figure 2 vs. Figure 4). Interestingly, there was no lag between the first and second adamantyl group hydroxylations for humans, indicating a highly coupled process. By contrast, mouse metabolism reflected poorer coupling for this pathway, requiring a much higher 5F-APINACA concentration to drive the reaction forward. In this case, 5F-APINACA-OH more readily undergoes release from the CYP, and must rebind for the second reaction to occur. The differences in the resulting metabolite structures are relatively minor, such that the kinetic differences may not alter activity toward CB1R and/or clearance significantly. The cause for the respective mechanisms seem to be due to the impact of the effector site on metabolism. The specificity of this effector site for other molecules, such as co-administered drugs, both controlled and otherwise, would lead to important impacts in response to 5F-APINACA use that would not conform to traditional, simple competitive inhibition models for predicting effects. Moreover, the presence and impact of this effector site could be a more common feature in SCB metabolism given the high conservation of structure among family members [38]. Nevertheless, for 5F-APINACA, there is high conservation in the overall metabolic clearance of the SCB between humans and CD-1 mice.

### 4.3. Similarities and Differences in CYPs Responsible for 5F-APINACA Metabolism Exist between Species

The introduction of phenotypic chemical inhibitors to metabolic reactions revealed the overall importance of CYPs in 5F-APINACA metabolism between species. For humans and CD-1 mice, CYPs were solely responsible for the metabolic reactions observed in this study, which justified normalizing the reactions to CYP concentrations over total protein content. For human liver microsomes, CYP3A4 dominated metabolism for both pathways, with minor contributions from CYP2C8, 2C19, and 2D6. These results are consistent with previously reported studies using recombinant CYPs [27]; however, the quantitative analyses performed in this study provided a critical scaling of their relative importance in metabolism. For the CD-1 mouse microsomal reactions, the human CYP phenotypic inhibitors implicated that Cyp3a11, along with Cyp2c37, Cyp2c50, and Cyp2c54, contributed equally to adamantyl hydroxylation, but Cyp3a11 was more efficient at oxidative defluorination vs. the Cyp2c isozymes. The more significant role of mouse Cyp2c isozymes in metabolism differed from that for humans. This observation could reflect the overlapping specificities of the mouse isozymes with Cyp3a11. For mice, the traditional CYP3A4 substrates, such as midazolam, are metabolized by both Cyp3a11 and Cyp2c isozymes (reviewed in [37]). It is then reasonable that Cyp3a11 and Cyp2c isozymes are similarly important in the overall metabolic clearance of 5F-APINACA. Moreover, the conserved role for CYP3A between species would lead to the similar regulation of metabolic activities. This would not be the case for Cyp2c isozymes in mouse reactions, and would lead to potential species differences, impacting the translatability of metabolism-dependent effects on 5F-APINACA response in mice to humans. Further in vitro and/or in vivo studies are necessary to determine whether these possibilities are significant enough to impact outcomes.

### 4.4. Limitations of the Current Findings

The insights gained from this work reflect three limitations. First, the current experimental design only minimized secondary metabolism due to the limits of absorbance sensitivity for metabolite quantitation. MS methods are more sensitive, yet require authentic standards that are not available. Moreover, it may not be possible to uncouple the sequential adamantyl oxidations. The reactions may arise from metabolic switching [39], in which the substrate rotates in the active site without being released from the active site, thus rendering dihydroxylations unavoidable. Second, the normalization to total CYP concentration obscures the role of the CYP subpopulation carrying out the reaction; however, this approach is more accurate than the current standard normalization to total protein. Lastly, another limitation is the reliance on phenotypic inhibitors for human CYPs to assess the role of individual mouse Cyps. Substrate specificities may differ among the CYPs, leading to ambiguity in the identification of isozymes participating in metabolic reactions. Despite the absence of mouse-specific inhibitors, the presence of orthologs between species [37] provides a degree of support for the notion that mouse Cyp isozymes in 5F-APINACA metabolism are similar to those for humans.

## 5. Conclusions

For the first time, we show that human and CD-1 mouse liver microsomes demonstrate comparable metabolic clearances for the SCB, 5F-APINACA. This effort was made possible by optimizing experimental methodology, thus limiting substrate turnover to yield more accurate kinetic parameters for metabolism, and the analysis of different kinetic modeling approaches. These analyses provide a more granular understanding of the mechanisms determining 5F-APINACA turnover. Furthermore, the collective findings of this work support the potential suitability of CD-1 mice to mirror human metabolism, and may provide insight into human metabolic and clinical responses following 5F-APINACA exposure. Moreover, factors impacting 5F-APINACA metabolism through Cyp3a11 would be similar to humans, given the similarity of this isozyme to human CYP3A4. Nevertheless, the potential contributions of Cyp2c enzyme(s) may confound the interpretation of these effects. Further research into how factors impact metabolism by CD-1 mice and humans are necessary to advance our understanding of the translatability of outcomes in mice toward humans for controlled substances such as SCBs.

## Figures and Tables

**Table 1:** Steady-state kinetics for 5F-APINACA metabolism by pooled human liver microsomes ^a^.

Metabolite	Model	V_max_, pmol min^−1^ mg protein^−1^	K_m_, µM	K_i_, µM	V_max_/K_m,_ pmol min^−1^ mg protein^−1^ µM substrate^−1^
5OH-APINACA	Michaelis–Menten	465 (446–485)	6.7 (5.6–7.9)		69
5F-APINACA-OH	Substrate inhibition	310 (280–350)	3.8 (2.9–5.1)	360 (230–600)	82
5F-APINACA-(OH)_2_	Substrate inhibition	77 (70–86)	1.3 (0.9–1.9)	810 (420–2800)	59
Combined	Substrate inhibition	400 (330–510)	3.3 (1.9–6.0)	300 (150–790)	120

^a^ Reaction conditions and analysis including identification of the most probable kinetic mechanism, as described in the Materials and Methods. Fitted data are shown in Figure 2. Values in parentheses reflect 95% confidence intervals for fitted kinetic constants.

**Table 2:** Individual rate constants for steady-state 5F-APINACA metabolism by pooled human liver microsomes ^a^.

Metabolite	k_cat1_ min^−1^	K_m1_ µM	k_cat1_/K_m1_ mL min^−1^ nmol^−1^	k_cat2_ min^−1^	K_m2_ µM	k_cat2_/K_m2_ mL min^−1^ nmol^−1^
5OH-APINACA (P1)	k_3_ 1.10 (0.35)	((k_2_ + k_3_ + k_4_)/k_1_) 5.69 (0.35)	0.193 (0.015)	k_7_ 1.23 (0.1)	((k_6_ + k_7_ + k_8_)/k_5_) 181 (10)	0.0065 (0.0013)
5F-APINACA-OH (P2)	k_4_ 1.18 (0.05)	((k_2_ + k_3_ + k_4_)/k_1_) 5.69 (0.35)	0.207 (0.016)	k_8_ 0.17 (0.08)	((k_6_ + k_7_ + k_8_)/k_5_) 85 (5)	0.0009 (0.0001)
5F-APINACA-(OH)_2_ (P3)	k_11_ 40 ^b^	((k_10_ + k_11_)/k_9_) 82 (20)	0.49 (0.12)	k_13_ 40 ^b^	((k_12_ + k_13_)/k_9_) 58 (6)	0.69 (0.07)

^a^ Reaction conditions and analysis including identification of the most probable kinetic mechanism, as described in the Materials and Methods. Fitted data are shown in Figure 3. Values in parentheses reflect standard error for fitted kinetic constants. ^b^ Values were set equivalent and constant, as described in the Results.

**Table 3:** Steady-state kinetics for 5F-APINACA metabolism by CD-1 mouse liver microsomes ^a^.

Metabolite	Model	V_max_ pmol min^−1^ mg protein^−1^	K_m_ or K_h_ µM	h	K_i_ µM	V_max_/K_m_ or V_max_/K_h_ pmol min^−1^ mg protein^−1^ µM substrate^−1^
5OH-APINACA	Hill	690 (650–730)	5.4 (4.4–6.7)	1.4 (1.0–1.8)		128
5F-APINACA-OH	Substrate inhibition	310 (275–345)	1.7 (1.2–2.4)		280 (180–480)	180
5F-APINACA-(OH)_2_	Hill	88 (81–95)	3.1 (2.1–4.0)	2.3 (1.3–4.8)		28
Combined	Substrate inhibition	465 (330–730)	3.8 (1.5–9.6)		220 (74–1300)	122

^a^ Reaction conditions and analysis including identification of the most probable kinetic mechanism, as described in the Materials and Methods. Fitted data are shown in Figure 4. For the Hill equation, the specificity constant is approximated as V_max_/K_h_. Values in parentheses reflect 95% confidence intervals for fitted kinetic constants.

**Table 4:** Individual rate constants for steady-state 5F-APINACA metabolism by CD-1 mouse liver microsomes ^a^.

Metabolite	k_cat1_ min^−1^	K_m1_ µM	k_cat1_/K_m1_ mL min^−1^ nmol^−1^	k_cat2_ min^−1^	K_m2_ µM	k_cat2_/K_m2_, mL min^−1^ nmol^−1^
5OH-APINACA (P1)	k_3_ 0.89 (0.04)	((k_2_ + k_3_ + k_4_)/k_1_) 3.75 (0.21)	0.238 (0.017)	k_7_ 1.17 (0.05)	((k_6_ + k_7_ + k_8_)/k_5_) 122 (6)	0.0096 (0.0006)
5F-APINACA-OH (P2)	k_4_ 0.738 (0.017)	((k_2_ + k_3_ + k_4_)/k_1_) 3.75 (21)	0.197 (0.012)	k_8_ 0.010 (0.04)	((k_6_ + k_7_ + k_8_)/k_5_) 122 (6)	0.0008 (0.00033)
5F-APINACA-(OH)_2_ (P3)	k_11_ 5 ^b^	((k_10_ + k_11_)/k_9_) 199 (4)	0.0251 (0.0005)	k_13_ 5 ^b^	((k_12_ + k_13_)/k_9_) 9.9 (0.7)	0.504 (0.034)

^a^ Reaction conditions and analysis including identification of the most probable mechanism, as described in the Materials and Methods. Fitted data are shown in Figure 5. Values in parentheses reflect standard error for fitted kinetic constants. ^b^ Values were set equivalent and constant, as described in the Results.

## Data Availability

The data presented in this study are openly available in FigShare at https://doi.org/10.6084/m9.figshare.20520963.v2.

## Abbreviations

The following abbreviations were used in the manuscript: SCB, synthetic cannabinoid; CB1R, cannabinoid type 1 receptor; N-(1-adamantyl)-1-(5-pentyl)-1H-indazole-3-carboxamide (5F-APINACA/5F-AKB48); LC, liquid chromatography; MS, mass spectrometry; NADPH, nicotinamide adenine dinucleotide phosphate (reduced).
